# *16S rDNA* Sequencing-Based Insights into the Bacterial Community Structure and Function in Co-Existing Soil and Coal Gangue

**DOI:** 10.3390/microorganisms11092151

**Published:** 2023-08-24

**Authors:** Mengying Ruan, Zhenqi Hu, Qi Zhu, Yuanyuan Li, Xinran Nie

**Affiliations:** 1 Institute of Land Reclamation and Ecological Restoration, China University of Mining and Technology-Beijing, Beijing 100083, China; mengyingruan@student.cumtb.edu.cn (M.R.); xrnie5190@student.cumtb.edu.cn (X.N.); 2 China University of Mining and Technology, Xuzhou 221116, China; tb21160024b0@cumt.edu.cn; 3 National Engineering Laboratory for Lake Pollution Control and Ecological Restoration, State Environmental Protection Key Laboratory for Lake Pollution Control, Chinese Research Academy of Environmental Sciences, Beijing 100012, China; zhu.qi@craes.org.cn

**Keywords:** coal gangue, co-occurrence network, microbial community, bacterial functional potential prediction, phenotype prediction

## Abstract

Coal gangue is a solid waste emitted during coal production. Coal gangue is deployed adjacent to mining land and has characteristics similar to those of the soils of these areas. Coal gangue–soil ecosystems provide habitats for a rich and active bacterial community. However, co-existence networks and the functionality of soil and coal gangue bacterial communities have not been studied. Here, we performed Illumina MiSeq high-throughput sequencing, symbiotic network and statistical analyses, and microbial phenotype prediction to study the microbial community in coal gangue and soil samples from Shanxi Province, China. In general, the structural difference between the bacterial communities in coal gangue and soil was large, indicating that interactions between soil and coal gangue are limited but not absent. The bacterial community exhibited a significant symbiosis network in soil and coal gangue. The co-occurrence network was primarily formed by Proteobacteria, Firmicutes, and Actinobacteria. In addition, BugBase microbiome phenotype predictions and PICRUSt bacterial functional potential predictions showed that transcription regulators represented the highest functional category of symbiotic bacteria in soil and coal gangue. Proteobacteria played an important role in various processes such as mobile element pathogenicity, oxidative stress tolerance, and biofilm formation. In general, this work provides a theoretical basis and data support for the in situ remediation of acidified coal gangue hills based on microbiological methods.

## 1. Introduction

Coal gangue is a solid waste produced during coal mining, washing, and processing, accounting for about 10–20% of raw coal output [1]. According to incomplete statistics, China’s coal gangue accumulates 40 billion tons, accounting for 120 km^2^ or more, and continues to increase at speeds of 0.37–0.55 Gt per year and become the largest industrial solid waste among China’s emissions [2]. After weathering or leaching in water, the activities of harmful heavy metals and soluble salts in coal gangue were enhanced. Some of them dissolved and entered water bodies and soil after precipitation events via surface runoff or groundwater, causing water and soil pollution in the mining area. Pollution caused by the long-term accumulation of coal gangue has been recognized as a serious environmental problem by the major coal-producing countries. Therefore, coal mine environments have been studied intensively, and the geochemical distributions of Co, Cr, Cu, Fe, Mn, Ni, Pb, and Zn have provided information on polluted sediments from coal mining regions [3]. Coal gangue was responsible for the heavy metal pollution of surrounding soil [4]. Coal mining activities led to the serious pollution of heavy metals in agricultural soil in northern Bangladesh. The average concentrations of Ti, Mn, Zn, Pb, As, Fe, Rb, Sr, Nb and Zr exceeded the world normal averages [5].

Although the environmental conditions are harsh in coal gangue, many microorganisms thrive in this habitat. In coal mining areas, the stacked coal gangue occupies large soil sheets and forms a new environment for microbial growth interfacing with the underlying soil. Bacteria play key roles in soil and coal gangue, e.g., in nutrient cycling and heavy metal migration [6,7,8,9]. Typically, a lot of bacteria are found on the surface of coal gangue. However, due to the particularities of coal gangue, long-term deposition and erosion are common [10]. At the interface of coal gangue and soil, rainfall facilitates bacterial growth and bacteria are found in both soil and coal gangue. The diversity, composition, and function of bacterial communities in coal gangue and soil are also different. Li et al. reported that coal-coating activities interfered with the soil bacterial community structure, resulting in a significant decrease in soil enzyme activity, and the microbiota was dominated by Proteobacteria, Actinomycetes, and Acidobacteria [11]. Ruan et al. studied the soil around a mine area and found seven dominant phyla. The relative abundance of Actinobacteria, Proteobacteria, and Gemmatimonadetes increased in the mine area. In contrast, the abundance of Acidobacteria, Planctomycetes, Bacteroidetes, and Chloroflexi decreased [12]. Sulfides such as yellow iron ore are oxidized under the influence of rainwater leaching and bacterial catalysis, resulting in the production of acid mine drainage (AMD) containing high concentrations of sulfate and heavy metal ions [13,14]. Microorganisms play an important role in the formation of AMD. Hu et al. showed that *Thiobacillus ferrooxidans*, *Thiobacillus thiooxidans*, and other oxidizing bacteria exacerbated the acidification of gangue [15]. Microbial groups around mine waters and mining areas have been reported frequently, but bacterial communities of coal gangue and the underlying soil environment have not yet been systematically studied. Most studies have focused on a single system. Microorganisms usually form ecological clusters or modules [16]. Such ecological clusters have multiple effects on pollutants in coal gangue and soil and provide important ecological services. To better understand the microbial diversity within habitats, it is essential to investigate the ecological network of bacterial communities in coal gangue and soil. There are few reports on functional and phenotypic analyses of microorganisms in coal gangue and underlying soil. Therefore, it is essential to perform functional and phenotypic analyses of microorganisms.

To fill this gap in the scientific literature, we used Illumina MiSeq high-throughput sequencing to explore the bacterial diversity and community of soil and coal gangue and studied the co-occurrence patterns of bacterial communities. The bacterial function and phenotype were predicted based on the Phylogenetic Investigation of Communities by Reconstruction of Unobserved States (PICRUSt) technique [17,18]. Three questions were addressed. (1) Do soil and coal gangue bacteria differ in diversity and community structure? (2) Do ecological networks and bacterial communities differ between coal gangue and soil? (3) What are the functions and phenotypes of bacteria in soil and coal gangue?

## 2. Materials and Methods

### 2.1. Study Area, Field Sampling, and Chemical Analysis

Sampling was performed in the Dongqu mining area in Gujiao City, Shanxi Province, in May 2021 (Figure S1). The region belongs to the temperate continental monsoon climate with approximately 4.2–14.2 °C annual mean temperature and 358–621 mm annual mean precipitation. A total of 23 samples of newly produced and weathered coal gangue were collected. Weathered coal gangue has been weathered for three years. The newly produced coal gangue has been dug up for a month. A total of 11 samples were newly produced coal gangue (X-1), and 12 were weathered coal gangue (S-1). Nine soil samples were collected adjacent to newly discharged gangue, including three samples adjacent to the gangue (YS), three near the gangue pile (NS), and three within the surrounding agricultural soil (MS). Samples were frozen in the field with liquid nitrogen and stored at −80 °C in the laboratory until DNA extraction. The coal sample was crushed to the particle size of flotation feed material below 0.5 mm. The characteristics of the samples are shown in Tables S1 and S2.

### 2.2. DNA Extraction, PCR, and Sequencing

We used the OMEGA kit (E.Z.N. A™ Mag-Bind Soil DNA Kit) to extract DNA. The Qubit 3.0 DNA detection kit was used to accurately quantify genomic DNA to determine the amount of DNA added to the PCR reaction. The primers used in PCR were fused with the V3–V4 universal primers of the sequencing platform. The initial PCR step was performed with the universal V3–V4 primers Nobar_341F (5′-CCTACGGGNGGCWGCAG-3′) and Nobar-805R (5′-GACTACHVGGTATCTAATCC-3′) [19]. The PCR reactions were performed in triplicate. A second round of amplification introduced Illumina bridge PCR compatible primers. It was detected by 2% agarose gel electrophoresis. To obtain a uniform long cluster effect and high-quality sequencing data, a Qubit 3.0 fluorescence quantifier was used to determine the library concentration. The PCR products were pooled in equal quantity for purification and sequenced on the Illumina MiSeq platform (Shanghai Sangon Biotechnology Co., Ltd., Shanghai, China) run using single reads. The QIIME v2.7 pipeline was mostly used for data quality controls and analyses. Operational taxonomic units (OTUs) were clustered with a 97% similarity threshold using UPARSE v7.1. We removed chimeras using VSEARCH. Singletons and doublets were not considered [18,20]. Using the SILVA 132 database, high-quality sequences were identified via RDP classifiers. The raw sequence data reported in this paper have been deposited in the Genome Sequence Archive (Genomics, Proteomics & Bioinformatics 2021) in the National Genomics Data Center (Nucleic Acids Res 2021), China National Center for Bioinformation/Beijing Institute of Genomics, Chinese Academy of Sciences.

### 2.3. Prediction of Microbiome Phenotypes and Potential Bacterial Functions

Predictions of the *16S rRNA* metataxonomics functional content were performed using the PICRUSt technique. Following quality control, the sequences were clustered into an OTU matrix by UCLASS with an identity threshold of 97% according to the standard database of PICRUSt. Taxonomic positions of representative sequences were annotated with the Greengenes database. The PICRUSt software was used to predict the functional potential of the bacterial community in samples. We then uploaded the OTU matrix to the BugBase website (https://bugbase.cs.umn.edu/) for microbial group prediction.

### 2.4. Statistical Analysis

The Illumina MiSeq high-throughput sequencing software calculated the community richness index (Chao1 and Ace Estimator) and community diversity indices (Shannon and Simpson index). The original data were organized with Excel 2016 and analyzed in the R software. The software Origin was used to draw charts. Venn diagrams were drawn with the R package ‘UpsetR’. Collinear diagrams were drawn with the R package ‘circlize’. Co-expression network graphs were drawn with the package ‘Ggraph’. The R ‘Gplots’ package was used for function prediction heat maps. Principal component analysis (PCA) was performed using Canoco 5.0. Data analysis and plotting were carried out with the R package ‘pheatmap’.

## 3. Results

### 3.1. The Diversity and Community Composition of Coal Gangue and Associated Soil Bacteria Were Different

**Figure 1:**
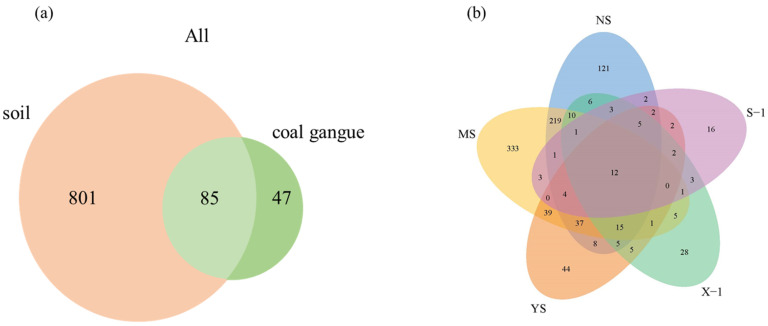
Venn diagram illustrating shared and unique OTUs in each type of sample. (**a**): soil and coal gangue; (**b**): five samples; X-1: newly produced coal gangue, S-1: weathered coal gangue, YS: Soil samples adjacent to the gangue, NS: soil samples near the gangue pile, MS: surrounding agricultural soil.

After quality filtering and the elimination of chimeric sequences, a total of 221,600 high-quality sequences (based on *16S rDNA*) were identified, and 933 OTUs were clustered from the entire sequencing dataset of all samples. The rarefaction curves for the bacterial OTUs obtained using this sequencing depth were sufficient to represent the metacommunity (Figure S2). The similarity analysis is shown in Figure S3. The microbial diversity index is shown in Table 1. The alpha diversity index was calculated based on the OTU. In coal gangue samples, the OTU richness ranged between 57 and 102, the Shannon index ranged between 1.58 and 3.65, the Chao index ranged between 58.5 and 102, the Ace index ranged between 63 and 102, the Simpson index ranged between 0.03 and 0.56, and the Shannon evenness ranged between 0.34 and 0.90. In soil samples, the OTU richness ranged between 181 and 681, the Shannon index ranged between 2.71 and 5.19, the Chao index ranged between 181.33 and 698.03, the Ace index ranged between 181.79 and 692.15, the Simpson index ranged between 0.02 and 0.26, and the Shannon evenness ranged between 0.52 and 0.80. Most bacterial systemic types (OTUs) existed in soil samples. The bacterial community structure in different types of samples was further analyzed at the OTU level (Figure 1). Of the 933 OTUs found in all samples, 85 were present in all types of samples (Figure 1a). Among them, there are seven core OTUs, which are present in all samples with relative abundance > 0.50%, including OTU3, OTU4, OTU1, OTU2, OTU10, OTU9, and OTU46. There were a large number of core bacterial communities in different types of samples, and they were mainly subordinate to the five genera including *Acinetobacter, Burkholderia*, *Sphingomonas, Staphylococcus*, and *Streptophyta*. Most of the OTUs were unique to different types rather than shared between them (Figure 1).

**Figure 2:**
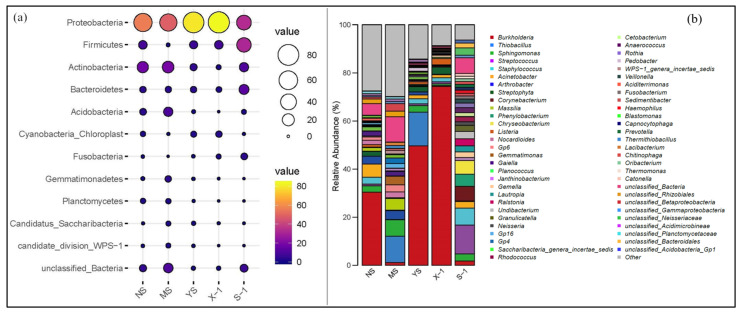
The bacterial distribution and relative abundance of different samples: (**a**) bacterial phylum; (**b**) bacterial genus. X-1: newly produced coal gangue, S-1: weathered coal gangue, YS: soil samples adjacent to the gangue, NS: soil samples near the gangue pile, MS: surrounding agricultural soil.

The bacterial community structure of different samples was further analyzed (Figure 2). A total of 11 bacteria were detected in all samples, of which five were the main bacteria, including Proteobacteria, Firmicutes, Actinobacteria, Bacteroidetes, and Acidobacteria (Figure 2a). In all samples, Proteobacteria dominated bacteria with a relative mean abundance of 60.89%. The relative abundance of Firmicutes was very high in weathered coal gangue samples. Planctomycetes and Candidate_division_WPS-1 were found in soil samples only. The abundance of Acidobacteria in newly discharged coal gangue samples was the lowest. At the genus level, 48 bacterial genera were identified (Figure 2b), and eight were the dominant bacterial genera, namely *Burkholderia*, *Thiobacillus*, *Sphingomonas*, *Streptococcus*, *Staphylococcus*, *Acinetobacter*, *Arthrobacter*, and *Streptophyta*. The relative abundances of *Burkholderia* were higher in X-1, YS, and NS but lower in MS and S-1. In contrast, Thiobacillus, Sphingomonas, and *Acinetobacter* relative abundances were higher in soil samples than in coal gangue samples. The relative abundances of *Streptococcus*, *Corynebacterium*, and *Staphylococcus* were higher in coal gangue samples than in soil samples.

### 3.2. Coal Gangue and Associated Soil Bacteria Showed Different Ecological Network Patterns

**Figure 3:**
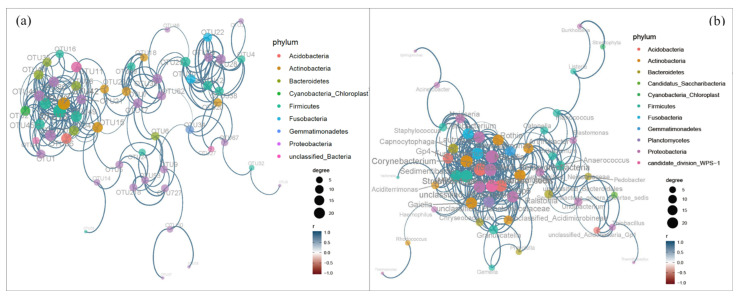
The co-expression network analysis of OTUs (**a**) and genus (**b**). X-1: newly produced coal gangue, S-1: weathered coal gangue, YS: soil samples adjacent to the gangue, NS: soil samples near the gangue pile, MS: surrounding agricultural soil.

A network analysis was performed based on all OTUs and biological genera (Figure 3). The soil and coal gangue samples networks differed in the number of nodes and edges. The OTU meta-network consisted of 55 nodes connected by 192 edges. The genus meta-network had 51 nodes connected by 248 edges. The number of positive correlations was higher than that of negative correlations in all networks. The 16S network was primarily occupied by Proteobacteria, Firmicutes, and Actinobacteria.

The node-level topological features of taxa were examined (Table 2). The frequency of species co-existence in the communities was not low. The average path length indicated that real networks usually had the small-world feature. Considering the network-level topological features, the bacterial network exhibited a high characteristic connectance and average degree. The bacterial network was complex with the OTUs interconnected. The two networks showed no scale, indicating that the organization of soil and coal gangue samples’ ecological networks was non-random. The connectivity between biological groups were very close.

### 3.3. Prediction of Bacterial Functional Potential

**Figure 4:**
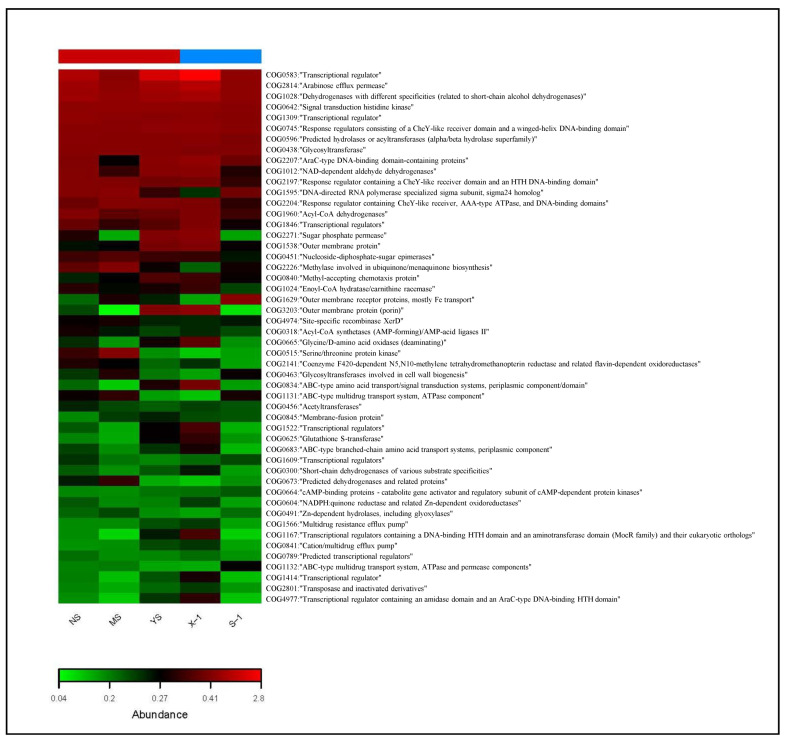
Functional prediction heat map of bacterial communities. X-1: newly produced coal gangue, S-1: weathered coal gangue, YS: soil samples adjacent to the gangue pile, NS: soil samples near the gangue pile, MS: surrounding agricultural soil.

To better understand the role of bacteria, further analyses were performed in the Cluster of Orthologous Groups (COG) database, and PICRUSt was used to predict the functional potential of bacteria in the samples. As a result, 50 COG functional categories were identified as well as 7942 functional proteins or enzymatic COGs (Figure 4). Some COGs were related to metal detoxification and organic degradation, such as transcription regulators (COG0583, COG1309, COG1846, COG1522, COG1609, COG1167, COG4977, COG1414, and COG0789) (Figure 4). Arabinose efflux permease (COG2814) and dehydrogenases (COG1012, COG1028) were relatively abundant.

**Figure 5:**
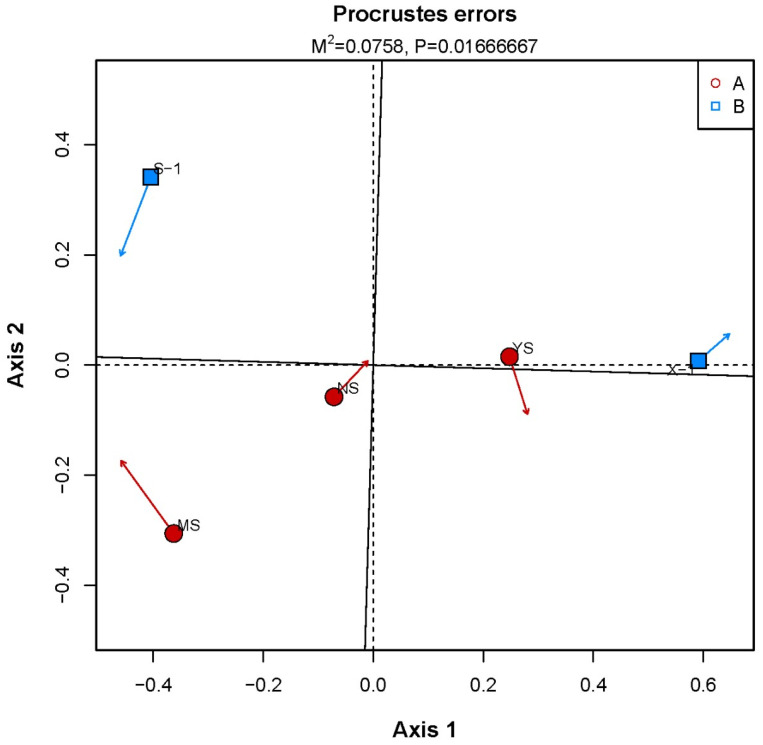
The relationship between species and function. A: soil samples; B: coal gangue samples. X-1: newly produced coal gangue, S-1: weathered coal gangue, YS: soil samples adjacent to the gangue, NS: soil samples near the gangue pile, MS: surrounding agricultural soil.

A principal component analysis (PCA) was performed based on COG functional annotations and genus–species classifications (Figure 5). The Procrustes error was M^2^ = 0.0758, indicating a strong correlation between the abundance composition of bacterial genera and the abundance composition of functional genes. The weathered coal gangue samples had the lowest similarity with other types. However, the closer the soil samples were to the coal gangue samples, the higher the similarity to the newly discharged gangue. There were also differences between newly discharged and weathered coal gangue samples.

**Figure 6:**
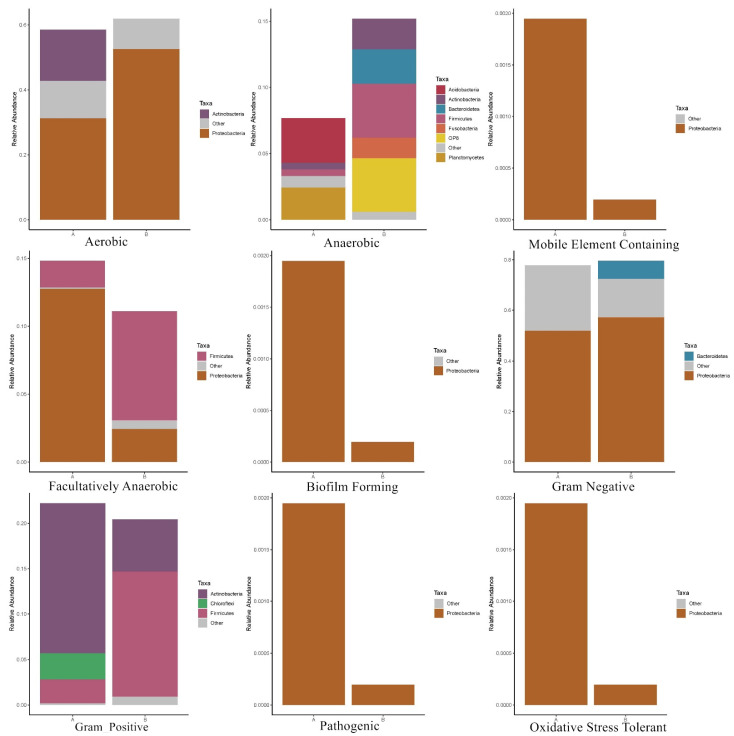
Nine phenotypic or functional prediction results. (A: soil; B: coal gangue.). X-1: newly produced coal gangue, S-1: weathered coal gangue, YS: soil samples adjacent to the gangue, NS: soil samples near the gangue pile, MS: surrounding agricultural soil.

BugBase classified the microbial communities according to seven phenotypes: Gram-positive, Gram-negative, biofilm-forming pathogenic, mobile element containing, oxygen utilizing (aerobic, anaerobic, facultatively anaerobic), and oxidative stress-tolerant bacteria (Figure 6). In soil samples, Proteobacteria plays a dominant role in the aerobic group, which is followed by Actinomyces. The dominant bacteria in the anaerobic group were different, particularly within the soil, and consisted of the Acidobacteria and Planctomycetes. The dominate taxa in coal gangue samples consisted of Actinobacteria, Bacteroidetes, Firmicutes and OP8. In the mobile element containing pathogenic, oxidative stress-tolerant, and biofilm-forming phenotypes, the dominant bacteria were Proteobacteria, which were more abundant in soil samples than in coal gangue samples. In the facultatively anaerobic group, soil samples were dominated by Proteobacteria and coal gangue samples were dominated by Firmicutes. In the Gram-negative group, proteobacteria were dominant, and the abundance of Bacteroidetes was higher in coal gangue than in soil samples. In the Gram-positive group, the abundance of Actinobacteria was higher in coal gangue than in soil samples, while the abundance of Firmicutes was lower.

## 4. Discussion

Soil and coal gangue are two distinct but related habitats for bacterial survival [21]. Coal gangue has characteristics similar to those of the soils in mining areas. Coal gangue and soil are relatively similar in their elemental, chemical, and mineral composition [22]. In this study, we systematically compared the diversity, composition, and symbiotic patterns of bacteria in coal gangue and adjacent soil and predicted the function and phenotype of bacteria. The bacterial communities of soil and coal gangue showed different community structures. The diversity of bacteria in soil was higher than that in coal gangue, and the closer the soil was to coal gangue, the lower the bacterial diversity. Coal gangue is poor in nutrients [23], its organic matter is highly solidified [24], its decomposition conversion rate is not high [25], and it does not have effective carbon sources [26]. The habitat conditions for microorganisms are, thus, poor, leading to a low bacterial diversity on the surface of coal gangue. The high diversity and variability in bacterial community composition and function play an important role in ecosystem structure [27,28]. Proteobacteria were the dominant bacteria in soil and coal gangue. Proteobacteria distribution is related to high carbon utilization [29]. Proteobacteria play a key role in the carbon cycle and have prominent eutrophication properties [30]. Acidobacteria are mostly distributed in poor soils with the ability to degrade complex and recalcitrant carbon compounds. [31]. Therefore, the abundance of Acidobacteria in the newly mined coal gangue was lower than that in soil. With the passage of time, the abundance of Acidobacteria in weathered coal gangue increases. Planctomycetes are widespread in soil and part of the microbial soil community [32]. The abundance of *Burkholderia* was higher in the soils near to gangue and in newly discharged gangue. *Burkholderia* is used for biological control, bioremediation, and plant growth promotion [33]. *Thiobacillus* belongs to the beta-subclass of the Proteobacteria [34] and is found on the sulfide mineral pyrite, marcasite, and arsenopyrite [35]. Over time, the activity of some acidophilic bacteria (such as *Thiobacillus*) increases, but these bacteria usually do not form a dominant genus at the beginning of the coal gangue oxidation process. Therefore, the emergence of *Thiobacillus* species occurs after a period of oxidation of coal gangue, which means that the advantage of *Thiobacillus* in the microbial community may indicate that the oxidation process of coal gangue has entered an accelerated cycle stage. The initial acidification of coal gangue may be due to the oxidation of ferrous ions promoted by *Thiobacillus*. *Sphingomonas* degrades refractory pollutants and can secrete useful gellan exopolysaccharides as a bacterial antagonist of plant pathogenic fungi [36]. The survival of *Streptococcus* depends largely on pH [37], and it is distributed in alkaline environments. *Staphylococcus* has potential applications in the bioremediation of oil-based drilling fluid [38]. *Arthrobacter* is the most common genus of aerobic bacteria in soil. It can survive for a long time under harsh environmental conditions and is diverse in its metabolism and ecology [39]. The division of *Streptophyta* may be important for embryophytes of terrestrial habitats [40]. *Massilia* was isolated from soil [41] but was not found in gangue samples. Different bacterial communities play important roles in various biochemical cycles.

Co-occurrence networks are used to analyze patterns of microbial communities and explore relationships within microbial communities [42,43]. This study represents the first time that a symbiotic network was applied to soil and coal gangue bacterial communities to elucidate symbiotic associations. The microbiome is organized into a tightly connected network of modules. Proteobacteria were the core bacteria in the interaction between different types of samples in our study. Compared with other dominant bacteria, Proteobacteria have a wider niche and higher anti-interference ability and play a leading role in maintaining the stability of the bacterial community interaction network. Other nondominant bacteria can be used as diversified libraries to enhance the resistance of bacteria to stress and environmental interference [44]. The collinear relationship network of bacteria showed an obvious co-occurrence pattern between soil and coal gangue. The greater the complexity of the network, the more stable the mixed and interactive bacterial community, which improves the transfer efficiency of resources [45]. The larger the number of network nodes and connections, the greater the interconnections between different bacteria groups. Ecological networks have low stability under disturbance, while network stability is considered high when there is high connectivity, high complexity, and low modularity [46,47]. The higher the diversity of bacteria, the more likely a bacterial group is to establish a relationship with the neighborhood [48]. This indicates that environmental changes strongly impact the symbiotic bacterial community and its structural composition.

The phenotypic and gene function prediction of bacteria indicated that different types of bacteria had similar functions and phenotypes. The functional spectrum of predictive COG profiles of bacteria showed that transcriptional regulators were the most abundant functional category. Transcription regulators are related to cell information storage and processes [49]. The numbers of COG were different, but their functions may be the same. For example, the functions of COG1309, COG1846, COG1522, COG1609, COG1167, COG4977, COG1414, and COG0789 are transcription regulators. The presence of Arabinose efflux permeases provides predicted support for the effective removal of pollutants. Dehydrogenases with different specificities were also abundant, indicating that cells could make full use of organic compounds such as sugars and amino acids to perform the substrate oxidation–reduction reaction [50]. Signal transduction histidine kinase, DNA-directed RNA polymerase specialized sigma subunit, Acyl-CoA dehydrogenases, and predicted hydrolases or acyltransferases also showed high abundances. They are important catalysts for cell synthesis, decomposition, and metabolism [51]. Aminoacid metabolism and carbohydrate metabolism are vital to microorganisms [52]. In addition, to reveal the responses of functional traits, this study used BugBase to predict potential phenotypes. In the aerobic, mobile element containing pathogenic, oxidative stress-tolerant, and biofilm-forming phenotypes, the dominant bacteria were Proteobacteria. The phylum Proteobacteria includes a variety of metabolic species, most of which are facultative or obligate anaerobic and heterotrophic, and they are self-sufficient, relying on the energy of photosynthesis [53]. Most proteobacteria are forming biofilms made of lipopolysaccharides [54]. The phylum Proteobacteria belongs to Gram-negative bacteria and is the largest phylum of bacteria. Like mobile elements containing bacteria, Proteobacteria have a high niche preference. The degradability of β-proteobacteria is variable, and there are more pathogenic bacteria, which greatly increases the complexity of the occurrence of various related pathogens [55].

## 5. Conclusions

In this study, bacterial communities in coal gangue and adjacent soils were studied using *16S rRNA* high-throughput sequencing, symbiotic networks, statistical analysis, and microbial phenotype prediction. Soil’s bacterial community diversity was higher than that of coal gangue. The dominant bacteria were Proteobacteria, Firmicutes, Actinobacteria, Bacteroidetes, and Acidobacteria. *Burkholderia*, *Thiobacillus*, *Sphingomonas*, *Streptococcus*, *Staphylococcus*, *Acinetobacter*, *Arthrobacter,* and *Streptophyta* were the dominant bacterial genera. The bacterial community had a significant symbiosis network in soil and coal gangue. The co-occurrence network was dominated by Proteobacteria, Firmicutes, and Actinobacteria. The phenotypic predictions at the biological level indicated that aerobic bacteria had the highest abundance, while anaerobic and facultative anaerobic bacteria had lower abundances. Oxidative stress-tolerant bacteria reduce the potential risk of pathogenic bacteria. Gram-negative bacteria were more abundant than Gram-positive bacteria. Proteobacteria played an important role in various processes such as mobile element assimilation, pathogenicity, oxidative stress tolerance, and biofilm formation.

## Figures and Tables

**Table 1:** Characteristics of bacterial richness and diversity indices in different samples.

Sample	Shannon	Chao	Ace	Simpson	Shannon Even
**X-1**	1.58	102.00	102.00	0.56	0.34
**S-1**	3.65	58.50	63.00	0.03	0.90
**YS**	2.71	181.33	181.79	0.26	0.52
**NS**	4.28	452.67	452.33	0.10	0.70
**MS**	5.19	698.03	692.15	0.02	0.80

X-1: newly produced coal gangue, S-1: weathered coal gangue, YS: soil samples adjacent to the gangue, NS: soil samples near the gangue pile, MS: surrounding agricultural soil.

**Table 2:** Topological parameters of the network of soil and coal gangue samples.

Topological Parameters	OTUs	Genus
Number of edges	192	248
Number of vertices	55	51
Connectance	0.12929	0.19451
Average degree	6.98182	9.72549
Average path length	3.52391	2.89569
Clustering coefficient	0.55731	0.64739
Betweenness centralization	0.30392	0.09992
Degree centralization	0.20404	0.24549

X-1: newly produced coal gangue, S-1: weathered coal gangue, YS: soil samples adjacent to the gangue, NS: soil samples near the gangue pile, MS: surrounding agricultural soil.

## Data Availability

Not applicable.

## Supplementary Materials

The following supporting information can be downloaded at: https://www.mdpi.com/article/10.3390/microorganisms11092151/s1, Figure S1: Geographical location of Gujiao, Shanxi Province, China; Figure S2: Rarefaction curves of operational taxonomic units (OTUs) at a 97% sequence similarity level for the samples; Figure S3: Analysis of similarity; Table S1: Element composition of weathered coal gangue; Table S2: Soil characteristics.
