# Supplementary material for: 16S rDNA Sequencing-Based Insights into the Bacterial Community Structure and Function in Co-Existing Soil and Coal Gangue
Source: Microorganisms. 2023 Aug 24;11(9):2151. doi: 10.3390/microorganisms11092151 (PMC10536285; doi:10.3390/microorganisms11092151)
Supplement: Supplementary file 1 [file microorganisms-11-02151-s001.zip › microorganisms-2510964-supplementary.pdf]

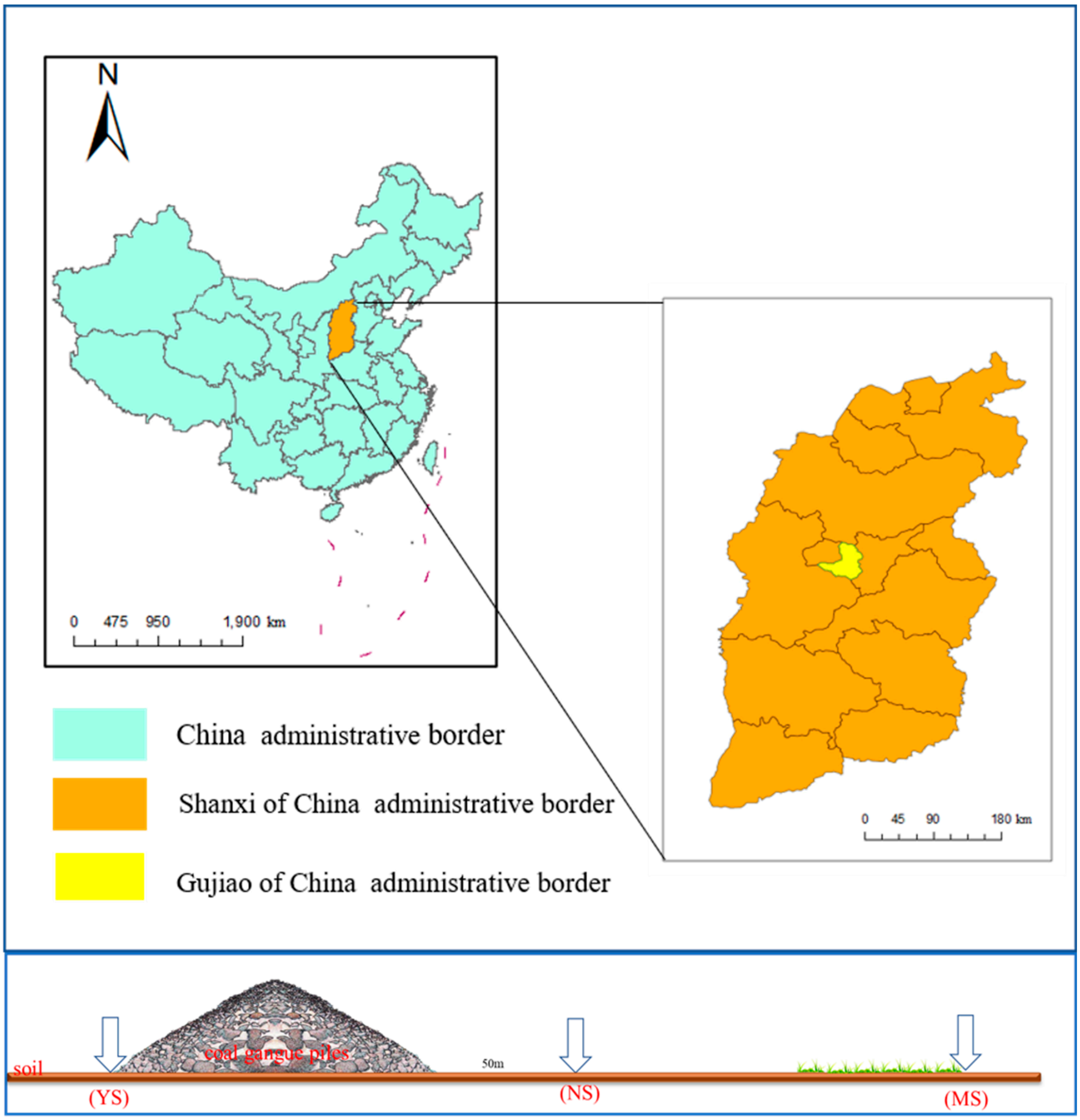

Figure S1. Geographical location of Gujiao, Shanxi Province, China

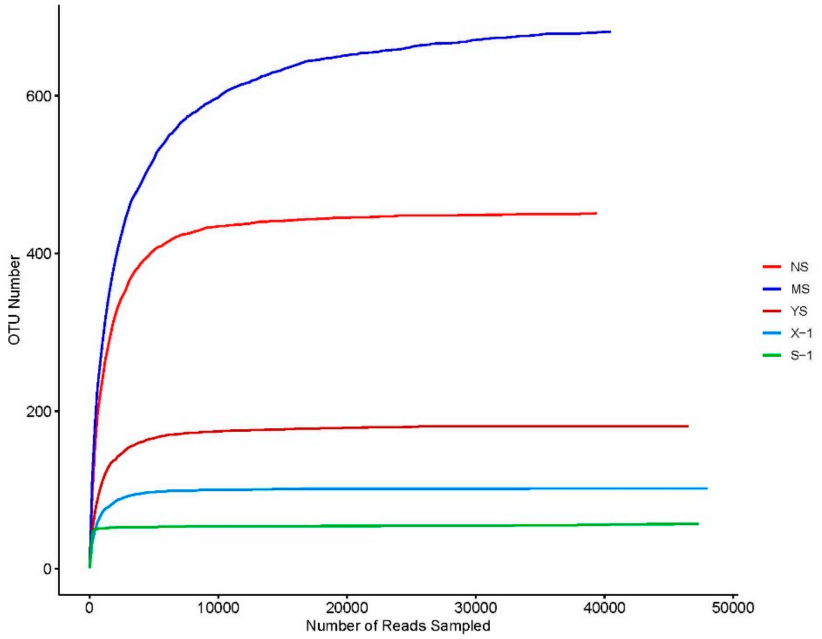

Figure S2. Rarefaction curves of operational taxonomic units (OTUs) at 97% sequence similarity level for the samples

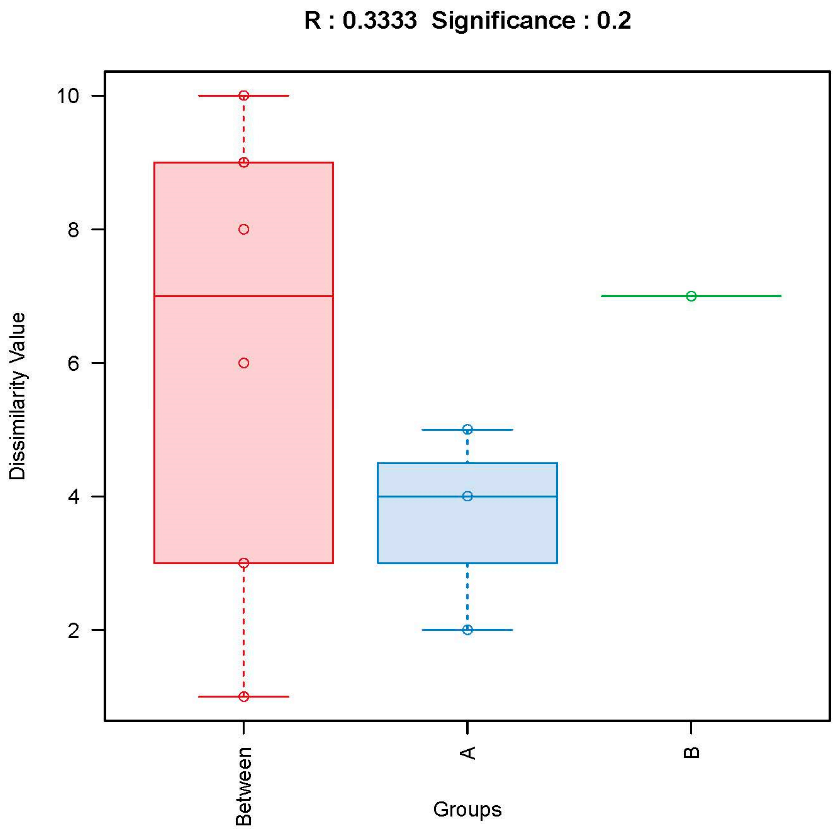

Figure S3. Analysis of similarities

Table S1 Element composition of weathered coal gangue

Element	Mn	Co	Ni	C	O	F	Mg	Al	Si	K	Ca	Fe
wt.%	0.07	0.04	0.04	7.17	55.88	0	0.27	15.68	18.34	1.49	0.35	0.66

Mn: Manganese; Co: Cobalt; Ni: Nickel; C: Carbon; O: Oxygen; F: Fluorine; Mg: Magnesium; Al: Aluminium; Si: Silicon; K: Potassium; Ca: Calcium; Fe: Iron;

Table S2 Soil Characteristics

	pH	SWC(%)	SOM(**g/kg**)	AK(**mg/kg**)	AP(**mg/kg**)	AN(**mg/kg**)
YS	**7.69±0.24**	**6.18±0.07**	**16.14±0.37**	**134.28±2.34**	**5.03±0.53**	**30.45±0.95**
NS	**7.43±0.05**	**7.91±0.05**	**21.09±0.51**	**158.95±2.87**	**9.74±0.84**	**40.27±0.75**
MS	**6.78±0.10**	**10.71±0.25**	**39.63±0.77**	**265.97±5.73**	**14.83±0.46**	**65.93±0.82**

SWC: soil water content; SOM: soil organic matter; AK: available potassium; AP: available phosphorus; AN: total nitrogen.
